# Beyond fresh: implications of produce prescription policy for food systems transformation

**DOI:** 10.1038/s41538-025-00704-4

**Published:** 2026-01-10

**Authors:** Carmen Byker Shanks, Bailey Houghtaling, Amy L. Yaroch

**Affiliations:** Center for Nutrition & Health Impact, Omaha, NE USA

**Keywords:** Environmental social sciences, Health care, Health humanities

## Abstract

This commentary examines a United States food policy which limits produce prescriptions to fresh fruits and vegetables within the Gus Schumacher Nutrition Incentive Program (GusNIP). We explore potential unintended consequences of this restriction and argue for expanding eligible types of fruits and vegetables to include frozen, canned, and dried options. A strategic policy revision for transforming food systems is provided, with insights that can be applied globally.

## Introduction

Food policy is implemented as a far reaching strategy to drive individual, systems, and environmental level changes. Yet, food policies, even when well intentioned, could reinforce dietary disparities and impede progress towards shaping equitable, resilient, and sustainable food systems^1^ The Food is Medicine movement in the United States (US), including approaches such as medically tailored meals, medically tailored groceries, and produce prescriptions, offers a timely platform to examine these dynamics. Food is Medicine interventions are offered in conjunction with healthcare systems to support patients with low-income to receive healthful food to prevent or manage chronic disease. Within this broader landscape, this commentary focuses specifically on produce prescriptions as a case through which to consider how policy design can support or constrain food systems transformation.

### The Gus Schumacher Nutrition Incentive Program (GusNIP) produce prescription policy

The 2018 Farm Bill allocated $250 million for the US Department of Agriculture’s (USDA) Gus Schumacher Nutrition Incentive Program (GusNIP) to support nutrition incentive projects, produce prescription projects, and the National Training, Technical Assistance, Evaluation, and Information Center (NTAE)^2^ These funds aim to improve food and nutrition security among Americans with low-income through incentives to purchase or acquire fruits and vegetables (FVs)^3^.

Specifically, the goal of the GusNIP Produce Prescription Program is to increase FV intake, reduce individual and household food insecurity, and lower healthcare utilization and associated costs. GusNIP grants fund Food is Medicine approaches to provide a prescription for *fresh* FVs within healthcare settings to individuals with low-income and chronic disease risk. Precise requirements for GusNIP produce prescription grants are transferred from Farm Bill policy into a Request for Applications (RFA), which have described eligible prescriptions as fresh, whole, or cut FVs without added sugars, fats or oils, and salt^4^ US Congress legislated the requirement at a time when many produce prescriptions were provided in conjunction with a farmers market partner, shaping the policy’s focus on fresh. Since then, the landscape has evolved, highlighting the need for policy adjustments to accommodate a broader range of produce types.

As one of the most well resourced produce prescription funding mechanisms, GusNIP’s *fresh* only requirement continues to shape how produce prescriptions are conceptualized and implemented in the US, even as some other Food is Medicine initiatives define produce prescriptions more broadly to include frozen, canned, and dried FVs. Notably, the GusNIP Nutrition Incentive (NI) program already allows fresh, frozen, canned, and dried fruits and vegetables that meet a “no added sugars, fats or oils, and salt” standard, demonstrating that broader eligibility can align with existing program criteria.

The NTAE, led by this Comment's co-authors, provides GusNIP grantees with evaluation and implementation technical assistance^5^ Through this work, the NTAE has identified potential unintended consequences of the GusNIP policy on food systems transformation, which currently limits produce prescriptions to *fresh* FVs. We outline how broadening eligible produce for prescriptions to include frozen, canned, and dried could better support equitable, resilient, and sustainable food systems. A policy revision strategy for transforming food systems is provided, with insights that can be applied globally.

### Unintended consequences of a GusNIP produce prescription policy

The requirement to limit prescriptions to *fresh* FVs may unintentionally constrain the effectiveness of the policy. This restriction could potentially lead to negative consequences for implementors and participants, as well as the broader food system, and may ultimately hinder progress toward GusNIP goals^4^

For grantees and partners implementing produce prescription interventions, limiting eligibility to *fresh* only may reduce their ability to provide an adequate amount and variety of FVs. GusNIP produce prescriptions are available at participating farm direct sites, clinics, and/or brick-and-mortar food retail sites. Settings that offer produce prescriptions must balance efforts for the public good with what is economically viable^6,7^ Stocking *fresh* FVs can be problematic for some retailers due to seasonality, food supplier(s), infrastructure, consumer demand, and capacity issues. Furthermore, many produce prescription projects aim to support local food producers that process *fresh* FVs into canned, frozen, and dried products^8^ However, without intentional procurement strategies, expanding eligible FV types could inadvertently increase reliance on large-scale supply chains rather than supporting local or regional food systems.

For produce prescription participants, evidence demonstrates that certain communities and geographies face distinct barriers to accessing affordable and available FVs, especially *fresh*^9–11^ *Fresh* only restrictions may disproportionately impact communities with limited retail infrastructure (e.g., rural) or shorter growing seasons. In these settings, frozen, canned, and dried FVs offer advantages such as convenience, lower cost, reduced preparation time, and reduced spoilage risk^12–14^ Frozen, canned, and dried FVs have extended shelf-life, which helps those with lack of transportation and/or inadequate kitchen equipment. Since frozen, canned, and dried FVs are preserved, they have longer shelf lives than *fresh*, which can help reduce spoilage and food waste in the household and across the food system. Restricting the types of FVs allowed may also limit an individual’s ability to satisfy foodways. For example, beans are a staple in many cultures, but restricting to only *fresh* has the potential to eliminate this option for those that only have access to canned or dried beans. For these reasons, participants’ ability to purchase and consume enough and a variety of FVs may be compromised; the opposite of what GusNIP aims to achieve.

The produce prescription RFA allows grantees to petition USDA for exemptions in cases when public health emergencies disrupt the food system or run counter to cultural food practices. Even so, grantees report apprehension to the NTAE about requesting an exemption or difficulty providing justification within stated guidelines. Moreover, grantees with limited resources and capacity may experience administrative burden to develop and submit such a request.

### Strategy for strengthening produce prescription policy and promoting food systems transformation

**Fig. 1:**
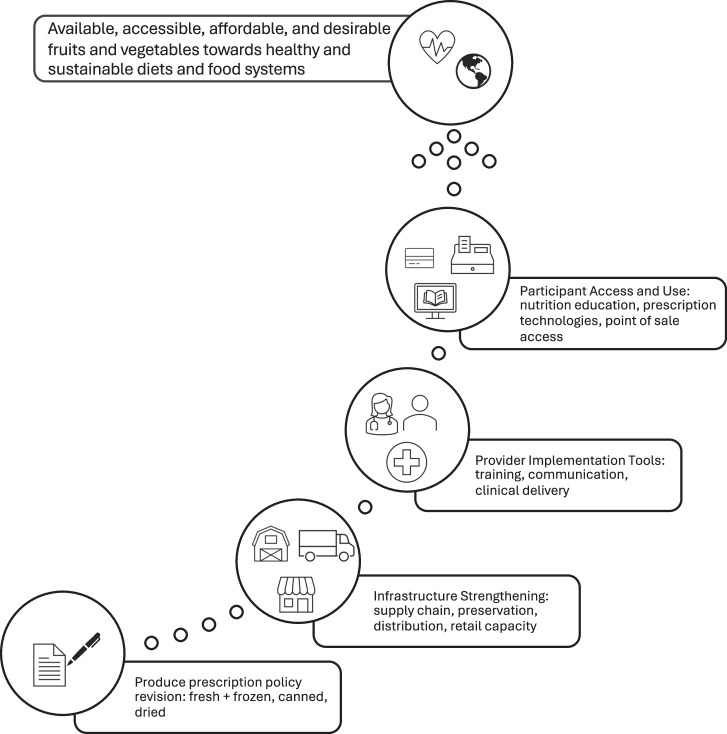
Strategy for transforming food systems through produce prescription policy revision.

It is critical to mitigate unintended consequences of food policy by revising or enhancing it in ways which remove impediments to food systems transformation. Within GusNIP, this likely means eliminating the *fresh* FV requirement and expanding to all relevant FV types, including fresh, frozen, canned, and dried. The strategy (Fig. 1) for advancing this food policy offers an opportunity to examine how these changes must occur alongside food system transformations, such as infrastructure development, provider implementation tools, and participant support, that collectively promote a sustainable and healthy food system.

A concrete policy revision may reduce produce prescription project implementation barriers and further support and bolster participant engagement^15^ Put simply, implementors of these projects could and would be more flexible in the type of FVs offered, and in the process, provide participants with greater variety. At the same time, expanding to include frozen, canned, and dried produce could also help address persistent challenges in the food system, such as limited access to healthy food, seasonality, and food waste. For example, frozen, canned, and dried FVs can be consistently accessible and available to participants in areas with limited access to healthy food or shorter growing seasons. Additionally, fresh produce that cannot be distributed in time or is imperfect can be preserved and sold, helping to increase profitability for farmers, support local economies, and reduce food waste.

To realize such benefits, it will be important to map out infrastructure across the food system that must be strengthened to support this Food is Medicine policy change. If all varieties of FVs (e.g., fresh, frozen, canned, and dried FVs) are included, the food system needs to be structured in a way that allows implementors to supply these foods and participants to access them. A resilient food system provides foods available through supply chain infrastructure, while Food is Medicine leverages these systems to deliver accessible, affordable, health-promoting foods. When intentionally aligned, they create a synergistic model for improving population health. Ongoing technical assistance and evaluation are essential to ensure that policy and systems changes are implemented effectively and appropriately. These supports can help identify and address challenges in real time, assess impacts across settings, and ensure continuous improvement toward intended outcomes.

Ensuring a consistent supply of varied FVs requires attention to both production and infrastructure that moves food through the system. While specialty crops already receive support through programs such as Specialty Crop Block Grants and subsidized crop insurance, these mechanisms do not consistently extend to the processing, aggregation, and preservation infrastructure needed to supply frozen, canned, and dried products. Strategic investment in regional preservation and processing capacity, supported in part by programs like the Local Agriculture Market Program (LAMP), could strengthen supply chain resilience and expand year-round FV access, though these programs have historically been underfunded. Strengthening supply chain logistics to ensure efficient handling, storage, and distribution of fresh, frozen, canned, and dried produce is also essential so that participants can reliably access items that meet their needs. Training and technical assistance will be necessary for implementers to adjust procurement and stocking practices when multiple produce forms are included, while nutrition education, participant-facing tools (e.g., apps, loyalty cards), and point-of-purchase prompts can support participants in selecting and using all types of produce. Simultaneously, effective produce prescription delivery requires provider-facing tools such as training, communication strategies, and clinical workflow support.

There are concrete ways in which policy change could be operationalized. The first step for a produce prescription policy in relation to GusNIP would involve government action, specifically through an act of Congress to change the legislation. The new US Farm Bill, where legislation for GusNIP resides, was expected to be passed in 2024, but is currently in limbo. There is some momentum for a change outlined in a bill by US Senators Cornyn, Luján, and Tuberville to allow frozen along with fresh^16^ If such a change were incorporated into the legislation, this would in turn influence the RFA language released by USDA. Beyond USDA’s administrative and logistical support, for example, the USDA could also play a pivotal role in promoting this information to potential GusNIP grant applicants. On the supply chain, USDA could support knowledge transfer and capacity building around sustainable practices with producers and other key actors. Other government agencies also have essential roles to play in the successful deployment of Food is Medicine policies. For instance, the FDA is responsible for overseeing food safety standards and labeling regulations, and is an entity that could ensure that any local produce used in these initiatives meets the necessary health and safety requirements. State and local agencies would also be crucial in supporting the on-the-ground implementation of Food is Medicine policies. For example, state level health and agriculture departments already coordinate produce prescription programs in some regions, and local health departments, Extension systems, and hospitals often facilitate participant enrollment, retailer partnerships, and nutrition education delivery. These agencies are directly involved in managing food system infrastructure, from coordinating local food hubs to facilitating access to nutrition incentives and produce prescriptions at the community level, including at food retail businesses. To avoid unintended shifts towards reliance on large-sclae supply chains, expanded eligibility can be paired with procurement guidance that encourages or prioritizes purchasing from local and regional producers when feasible. For this policy change to be successful, it will require a stepwise and coordinated effort across multiple government agencies.

It is also important to outline potential drawbacks of expanding eligibility to include frozen, canned, and dried produce. For example, expanding GusNIP’s eligibility could lead to local market distortions, with shifts in demand adversely affecting various types of produce pricing and availability. More likely, managing a broader range of produce introduces logistical complexities that could create administrative challenges for both implementers and participants, requiring adjustments to infrastructure, distribution systems, tracking, and consumer purchasing behaviors. Food safety, labeling, and quality control are already overseen through standard regulatory mechanisms in retail supply chains, suggesting that including frozen, canned, and dried FVs would not require substantial new oversight but rather alignment with existing standards. Moreover, some organizations may face increased administrative burdens as they adapt. However, challenges can be mitigated with strategic planning and investments alongside policy implementation. For example, the US government’s recent change in administration, which includes the Health and Human Services "Make America Healthy Again" initiative, could align with Food is Medicine efforts to support policy change. However, realizing these goals will likely require strengthened support and capacity within key US agencies, some of which have recently experienced funding reductions and operational constraints.

Efforts to empirically understand intended and unintended implications of Food is Medicine policy changes on equitable, resilient, and sustainable food systems are also warranted. Little empirical data beyond NTAE-level observations on the relationship between GusNIP policy specifications and impacts on key food and health and business/organizational outcomes exists, although co-authors are examining some of these questions with Robert Wood Johnson Foundation funding^17^ Tangential research about food-related behaviors involving Supplemental Nutrition Assistance Program (SNAP) participants found they are more likely to buy frozen FVs^18^ Another study found that purchasing frozen FVs is associated with higher overall FV intake in the general population^19^ This type of peripheral research is important when considering how Food is Medicine policy changes may impact participants and broader outcomes.

These insights are relevant beyond the US, as there is a growing global precedent for integrating food-based approaches into healthcare systems to support patients with low-income in accessing nutritious food to prevent or manage chronic diseases. For instance, the United Kingdom’s Healthy Start program provides pregnant women and families with children under four years old that have low-income with vouchers to purchase healthy foods, including FVs. Another example is Israel’s new pilot project launching in 2025 to test produce prescriptions for patients with diabetes and low-income. While each country has unique healthcare, policy frameworks, and food systems, it is crucial to assess the effectiveness of these initiatives in terms of equity, resilience, and sustainability.

In a world where food insecurity is persistent, FV intake are well below recommended levels, and diet-related chronic disease is at an all-time high, it is paramount that food policy supports food systems transformation through available, accessible, affordable, and desirable foods towards healthy and sustainable diets.

## Data Availability

The contents of this manuscript do not represent original research. Thus, no associated dataset is available.
